# Minnesota State Medical Society

**Published:** 1871-08

**Authors:** 


### MINNESOTA STATE MEDICAL SOCIETY.

The Semi-annual Meeting of this Society was held at Minneapolis, Minn., on Tuesday and Wednesday, June 13th and 14th.

The President, Dr. Franklin Staples, of Winona, delivered an eloquent and appropriate opening address, detailing the objects of the meeting, and congratulating the members on the prosperous condition of the Society, and the high position it had attained in the interests of the profession, as evinced by the large number of members present at the opening session.

About sixty members answered to their names on roll-call.

Dr. A. E. Ames and Dr. N. B. Hill, delegates of the meeting of the American Medical Association, held at San Francisco, May 2d, gave a very interesting account of their trip thither—the country, the meeting of the Association, and the manner in which they were entertained by the generous Californians.

Dr. B. Mattocks, of St. Paul, read a well-considered and able paper on the "Diseases of Children," which was discussed by Drs. Goodrich, Rhodes, Hill, Reiner, Chace, and Hewitt. Dr. Mattocks, in his essay, took the position that the nature and treatment of diseases of children was the same in principle as in the diseases of adults. This position was strongly contradicted by Dr. Hewitt, of Red Wing, who took the position that the child was an imperfect, immature being, whose pathology was entirely distinct from that of the conditions of human maturity.

A vote of thanks was passed for the essay, and it was referred to the Committee on Publication.

On Tuesday evening, the members of the Association and other invited guests, assembled at the residence of Dr. A. E. Ames, to partake of an elegant banquet given by the doctor in honor of the Association.

On Wednesday, Dr. A. J. Stone, editor of the *Northwestern Medical and Surgical Journal,* offered a draft of a bill to legalize dissection, to be presented for adoption by the Legislature. Dr. Stone moved that a committee of three be appointed to present the matter to the Legislature, and urge its passage. Considerable discussion followed, and several amendments were offered, but the original motion was finally carried. The committee, as approved by the President, consisted of Drs. A. J. Stone, J. H. Stewart, and C. P. Adams.

Dr. C. S. Sheldon, of Winona, was appointed essayist for the annual meeting, Dr. L. P. Hodge, of Farmington, as alternate.

Dr. W. W. Mayo, of Rochester, reported a case of rectocele of fourteen years duration, which was finally cured. Dr. C. G. Goodrich reported a case of ovarian cyst in a child of eight years.

All three of the gentlemen received the thanks of the Association for their papers, the same being referred to the Committee on Publication.

CONCLUSION.

Dr. Staples, the President, gave expression to the views of the Society in announcing that the members were delighted with this beautiful and promising city in which they had been so hospitably entertained. He was gratified at the large attendance, and bespoke for the next annual meeting a full attendance and an increased interest. The Society, on motion, adjourned *sine die.*

